# Morbid Obesity in Women Is Associated with an Altered Intestinal Expression of Genes Related to Cancer Risk and Immune, Defensive, and Antimicrobial Response

**DOI:** 10.3390/biomedicines10051024

**Published:** 2022-04-29

**Authors:** Ailec Ho-Plágaro, Cristina Rodríguez-Díaz, Concepción Santiago-Fernández, Carlos López-Gómez, Sara García-Serrano, Flores Martín-Reyes, Francisca Rodríguez-Pacheco, Alberto Rodríguez-Cañete, Guillermo Alcaín-Martínez, Luis Vázquez-Pedreño, Sergio Valdés, Lourdes Garrido-Sánchez, Eduardo García-Fuentes

**Affiliations:** 1 Instituto de Investigación Biomédica de Málaga-IBIMA, 29010 Malaga, Spain; ailec_hp@hotmail.com (A.H.-P.); cris.rdrz@gmail.com (C.R.-D.); conchisantiagofernandez@gmail.com (C.S.-F.); carlos.lopez@ibima.eu (C.L.-G.); garciasara79@hotmail.com (S.G.-S.); floresmarey@hotmail.com (F.M.-R.); paqui.endocrino@gmail.com (F.R.-P.); galcainm@hotmail.com (G.A.-M.); lourgarrido@gmail.com (L.G.-S.); 2 Unidad de Gestión Clínica de Aparato Digestivo, Hospital Universitario Virgen de la Victoria, 29010 Malaga, Spain; 3 Departamento de Biología Celular, Genética y Fisiología, Universidad de Málaga, 29010 Malaga, Spain; 4 Unidad de Gestión Clínica de Endocrinología y Nutrición, Hospital Regional Universitario de Málaga, 29009 Malaga, Spain; 5 CIBER de Diabetes y Enfermedades Metabólicas Asociadas (CIBERDEM), 29010 Malaga, Spain; 6 Unidad de Gestión Clínica de Cirugía General, Digestiva y Trasplantes, Hospital Regional Universitario, 29010 Malaga, Spain; arodriguezcane@hotmail.com; 7 Unidad de Gestión Clínica de Aparato Digestivo, Hospital Regional Universitario, 29010 Malaga, Spain; luichivazquez@hotmail.com; 8 Unidad de Gestión Clínica de Endocrinología y Nutrición, Hospital Universitario Virgen de la Victoria, 29010 Malaga, Spain; 9 CIBER de Enfermedades Hepáticas y Digestivas (CIBEREHD), 29010 Malaga, Spain

**Keywords:** microarray, duodenum, morbid obesity, inflammation, cancer, insulin resistance, immune system

## Abstract

Background: Little is known about the relation between morbid obesity and duodenal transcriptomic changes. We aimed to identify intestinal genes that may be associated with the development of obesity regardless of the degree of insulin resistance (IR) of patients. Material and Methods: Duodenal samples were assessed by microarray in three groups of women: non-obese women and women with morbid obesity with low and high IR. Results: We identified differentially expressed genes (DEGs) associated with morbid obesity, regardless of IR degree, related to digestion and lipid metabolism, defense response and inflammatory processes, maintenance of the gastrointestinal epithelium, wound healing and homeostasis, and the development of gastrointestinal cancer. However, other DEGs depended on the IR degree. We mainly found an upregulation of genes involved in the response to external organisms, hypoxia, and wound healing functions in women with morbid obesity and low IR. Conclusions: Regardless of the degree of IR, morbid obesity is associated with an altered expression of genes related to intestinal defenses, antimicrobial and immune responses, and gastrointestinal cancer. Our data also suggest a deficient duodenal immune and antimicrobial response in women with high IR.

## 1. Introduction

Obesity has reached epidemic proportions in recent decades. It is associated with numerous disorders such as a low-grade systemic inflammation, insulin resistance, dyslipidemia, and hypertension, which in turn increase the risk of heart disease, stroke, and type 2 diabetes mellitus (T2DM) [1,2]. Overfeeding, changes in the composition of the diet (higher caloric density), and a sedentary lifestyle are some of the main underlying causes for overweight and obesity [3]. The gastrointestinal (GI) tract is the first point of interaction between the host, the microbiota, and antigens coming from the diet; therefore, unhealthy food consumption habits disturb, in the first instance, the composition of the intestinal microbiota and the homeostasis of the GI tract. In this sense, it is known that obesity is associated with dysbiosis, altered intestinal mobility, permeability, and inflammation, as well as gut–brain disorders [4,5]. These imbalances can not only modulate the metabolism, promoting and aggravating the development of obesity, but can also be the starting point that triggers a series of events involved in the development of comorbidities associated with obesity, such as insulin resistance and T2DM [6].

Most studies on obesity and T2DM are focused on the study of metabolism modulating tissues such as adipose, skeletal muscle, or liver [7]. The remission of T2DM, as well as the improvement of other pathologies in patients with morbid obesity undergoing bariatric surgery, has revealed the important involvement of the small intestine in metabolism and body homeostasis [8]. However, the GI tract is not only involved in digestive functions. The intestine, through the secretion of specific molecules, and the immune system modulate the composition of the microflora, which is considered an important virtual endocrine organ [9]. On the other hand, the GI has a direct role in the regulation of the body’s energy balance through the coordinated action of hormonal, neural, and immunological signals [10]. Although studies have been conducted on the matter, little is known about the intestinal transcriptomic changes in patients with morbid obesity and how these changes may be different depending on the degree of insulin resistance. To date, there are few studies in this regard, with the majority being carried out in animal models [11,12,13,14]. In previous studies we have observed significant changes in the protein expression of various cytokines and chemokines related to pro/anti-inflammatory processes in the duodenum of patients with morbid obesity and different grades of insulin resistance, as well as in intestinal permeability [15]. Moreover, we have observed changes in the duodenal transcriptome of women according to their insulin resistance level, independently of the patient’s body mass index (BMI) [16].

To provide more information, in this study we aimed to identify intestinal genes that may be associated with the development of obesity regardless of the degree of insulin resistance of patients. To this effect, we identified genes with the same expression profile in the duodenum of women with morbid obesity alongside both high and low insulin resistance, which are the same genes that are associated with BMI regardless of insulin resistance.

## 2. Material and Methods

### 2.1. Subjects

The study included 15 patients with morbid obesity (MO) (BMI > 40 kg/m^2^) and 6 healthy, non-obese (NO) women (BMI < 30 kg/m^2^) (Table 1) (see Supplementary Table S1 for more detailed information about the women). The MO women included in this study underwent sleeve gastrectomy (SG) at the Regional University Hospital of Malaga (Spain). One month before SG, they underwent programmed gastroscopy to discard alterations in the stomach, at which time duodenal biopsies were obtained. Subjects were excluded if they had T2DM with anti-diabetic oral or insulin treatment, acute inflammatory disease, or infectious disease or if the patient did not consent. The MO women were classified into two groups according to the homeostasis model assessment of IR (HOMA-IR) level (low HOMA-IR value (<4.7) (MO-low-IR, n = 7) or high HOMA-IR value (>4.7) (MO-high-IR, n = 8)) (both groups without treatment for T2DM) [15,16]. The cut-off point for the HOMA-IR was taken from previous studies carried out in our population of patients with morbid obesity [15,16,17]. The non-obese women were selected among those who underwent programmed gastroscopy at the Virgen de la Victoria University Hospital (Málaga, Spain) with non-pathological results, at which time duodenal biopsies were taken. They had a similar average age to the MO group and reported that their body weight had been stable for at least 3 months prior to the study. We included only one group of NO patients who had low insulin resistance (NO-low-IR). This cut-off point for the HOMA-IR was obtained from the 75th percentile of the HOMA-IR value for non-obese subjects in our area with normal glucose metabolism according to the 1998 American Diabetes Association classification [18]. These subjects (NO and MO women) had been included in previous studies [15,16]. All individuals included were of Caucasian origin. The samples were processed and frozen immediately after arrival at the Regional University Hospital Biobank (Andalusian Public Health System Biobank). Participants gave their written informed consent. The study was carried out in accordance with the Code of Ethics of the World Medical Association (Declaration of Helsinki) and approved by the Malaga Provincial Research Ethics Committee, Spain (PI12/00338).

### 2.2. Analytical Procedures

Serum glucose, cholesterol, and triglycerides (Randox Laboratories Ltd., Antrium, UK) were measured in fasting state by standard enzymatic methods. Insulin was analyzed using an immunoradiometric kit (DIAsource ImmunoAssays SA, Louvain-la-Neuve, Belgium). The homeostasis model assessment of insulin resistance (HOMA-IR) was calculated with the following equation: HOMA-IR = fasting insulin (µIU/mL) × fasting glucose (mmol/L)/22.5 [17].

### 2.3. Duodenal Samples

Duodenal biopsies from NO and MO women from the 2nd part of the duodenum were obtained in a fasting state during a gastroscopy that the patients underwent [15,16]. The mucosa was washed with physiological saline solution, immediately frozen in liquid nitrogen, and maintained at −80 °C until analysis. Total RNA isolation from frozen duodenal biopsy samples was performed using an RNeasy Mini Kit (Qiagen GmbH, Hilden, Germany).

### 2.4. Microarray Procedure

The study of the differential expression profiling, with a SurePrint-G3 Human GE 8 × 60 K microarray kit (ID 028004; GPL13607) (Agilent Technologies, Madrid, Spain), was carried out following an experimental design in which a two-pairwise comparison was performed. Samples for each experimental condition were labeled, hybridized, washed, and scanned according to the two-color microarray-based gene expression analysis v6.5 protocol of the Genetic Diagnostic Bioarray facilities (Bioarray, Alicante, Spain). This microarray contained 62,976 probes, of which 58,717 had no controls. These probes correspond to 21,414 genes (filtered by gene identification). From these 58,717 probes, 45,283 showed mRNA expression. The data discussed in this publication have been deposited in the NCBI’s Gene Expression Omnibus (GEO) (GEO Series access number GSE147562).

### 2.5. Microarray Data Analysis

Data analysis was performed with Agilent Feature Extraction Software v.10.7 (Agilent Technologies), using the latest gene annotations available. The expression of each gene was reported as the ratio of the value obtained after each condition relative to the control condition after normalization of the data against the median of the control samples. A filter was applied to select the genes with significant differential expression (DEGs) that displayed an adjusted FDR of less than 0.05 by a nonparametric analysis (Rank Product). This analysis has the advantage of being less sensitive to the variability of the samples; thus, it is suitable in systems where high variability is expected. Prior to any further analysis, the array points were filtered in order to discard replicated genes, filtering first by probe name and later by systematic name. Due to the direction of the hybridizations, the DEGs in this study were those that were up- or downregulated in the MO groups when compared with the NO group. We obtained two lists of DEGs (FDR < 0.05) between (a) the MO-low-IR group with respect to the NO-low-IR group, and between (b) the MO-high-IR group with respect to the NO-low-IR group. These two gene lists were overlapped by a Venn diagram obtaining the upregulated and downregulated DEGs shared by MO-low-IR and MO-high-IR women with respect to the NO-low-IR group (genes in intersection), as well as the exclusive DEGs of MO-low-IR and MO-high-IR.

### 2.6. Functional Enrichment

The lists of up- and downregulated genes offered by the Venn diagram were functionally analyzed using Gene Set Enrichment Analysis (GSEA; GSEA/MSigDB web site v6.4 version; MSigDB database v7.2 updated September 2020; https://www.gsea-msigdb.org/gsea/index.jsp, accessed on 27 March 2021) [19,20], which is a computational method that determines whether an a priori-defined set of genes shows statistically significant, concordant differences between two biological states. We computed overlaps between our gene sets and gene sets in the MSigDB database. Using this platform, we identified gene sets significantly overrepresented (FDR *q*-value < 0.05) in different selected collections and sub-collections. The FDR *q*-value is a false discovery rate analog of hypergeometric *p*-value after correction for multiple hypothesis testing according to Benjamini and Hochberg. Overrepresentation analysis is a technique for determining whether a set of terms is present more than it would be expected. The collections and sub-collections used in the analysis were the following: gene ontology gene sets (GO terms), chemical and genetic perturbations (CGP), hallmark (H), human phenotype ontology (HPO), KEGG subset of canonical pathways (CP:KEGG), REACTOME subset of canonical pathways (CP:REACTOME), and cancer modules (CM). GO defines the function used to describe gene function with respect to three aspects: molecular function (MF) (molecular activities of gene products), cellular component (CC) (where gene products are active), and biological process (BP) (pathways and larger processes made up of the activities of multiple gene products).

### 2.7. Technical Validation of Microarray Data by Real-Time–Quantitative PCR (RT–qPCR)

The technical validation of the data obtained in the microarray was carried out in our previous study performed in these same patients [16].

### 2.8. Statistical Methods

We designed the experiment taking into account the anticipated number of undifferentially expressed genes in the microarray (20,000), 1 as the number of false positives, 0.80 as the desired power, 2 as the mean difference in log-expression between two groups, and 1 as the anticipated standard deviation of the difference in log-expression between two groups. With this design, the sample size for each group is 6 [21,22]. The statistical analysis was performed with R statistical software, version 2.8.1 (Department of Statistics, University of Auckland, Auckland, NZ; http://www.r-project.org/). Differences between groups were established using the Mann–Whitney test. Values were considered to be statistically significant when *p* ≤ 0.05. The results are given as the median (interquartile range). The statistical significance of the microarray gene expression is described above.

## 3. Results

### 3.1. Differentially Expressed Genes (DEGs) in the Microarray Hybridizations

**Figure 1:**
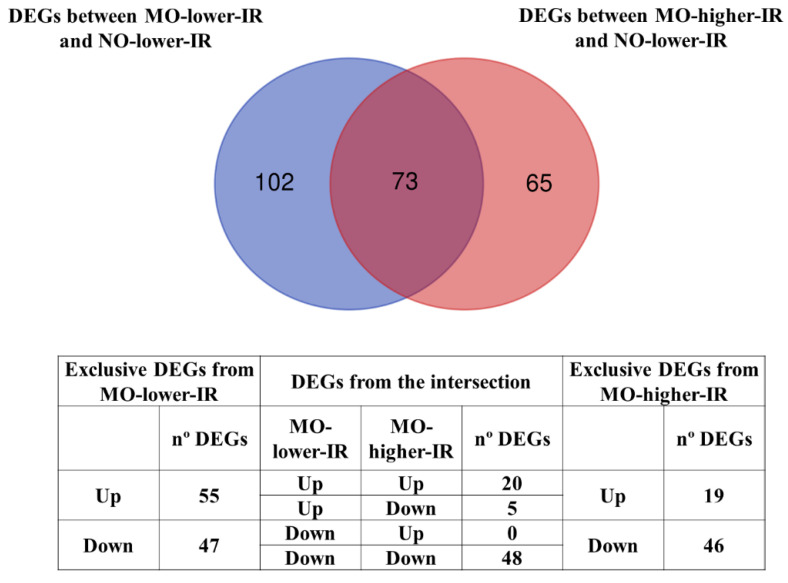
Venn diagram of the differentially expressed genes (DEGs) (FDR < 0.05) in the microarray. We obtained two lists of DEGs between the MO-high-IR and NO-low-IR groups and between the MO-low-IR and NO-low-IR groups. NO: non-obese women. MO: women with morbid obesity.

We obtained two lists of DEGs (FDR < 0.05): between the MO-low-IR and NO-low-IR groups and between the MO-high-IR and NO-low-IR groups. Excluding the unnamed genes, we found a total of 175 DEGs among the MO-low-IR and NO-low-IR groups (80 upregulated and 95 downregulated) and 138 DEGs between the MO-high-IR and NO-low-IR groups (39 upregulated and 99 downregulated). In the intersection of these two groups of DEGs, we found 73 DEGs that were associated with BMI, regardless of the degree of insulin resistance (Figure 1). It is worth noting the high degree of similarity in the up/downregulation patterns of these shared genes, where we found 20 upregulated and 48 downregulated genes in both groups of obese patients. We only found five genes shared between both groups (MUCL3, PGC, TCN1, TFF2, BPIFB1) that were upregulated in MO-low-IR but downregulated in MO-high-IR when compared with women without obesity (Figure 1).

However, other DEGs were differentially expressed between MO and non-obese groups that were dependent on insulin resistance degree; there were 102 DEGs exclusively in the MO-low-IR group and 65 DEGs exclusively in the MO-high-IR group with respect to the NO-low-IR group. In this case, we observed a greater presence of exclusive upregulated DEGs in MO-low-IR and a predominance of downregulated genes in MO-high-IR (Figure 1).

### 3.2. Functional Enrichment

With the aim of delving into the processes, routes, or biological aspects related to the set of differentially expressed genes, we selected a wide range of gene sets collected in The Molecular Signatures Database (MSigDB) for functional analysis with GSEA. The complete analysis of the overrepresented gene sets (FDR < 0.05) and the associated *p*-value and FDR *q*-value are represented in Supplementary Table S2. To simplify the information, we have grouped the gene sets into the most relevant biological functions, processes, or responses in which gene expression is upregulated and downregulated in the duodenum of women with morbid obesity (Table 2); selected gene sets for each function are indicated in Supplementary Table S2.

Our results indicated an alteration in important processes such as inflammation, immune response, hypoxia, digestion, maintenance of the gastrointestinal epithelium, wound healing, homeostasis, and cell proliferation; as well as in the response to biotic stimuli, drugs, xenobiotics, and toxic substances, among others (Table 2). On the other hand, we found the dysregulation of a group of genes that are associated with the development of gastrointestinal cancer. A more detailed analysis of the most relevant results obtained on the processes and biological functions is shown in the following sections.

#### 3.2.1. Digestion and Lipid Metabolism

**Figure 2:**
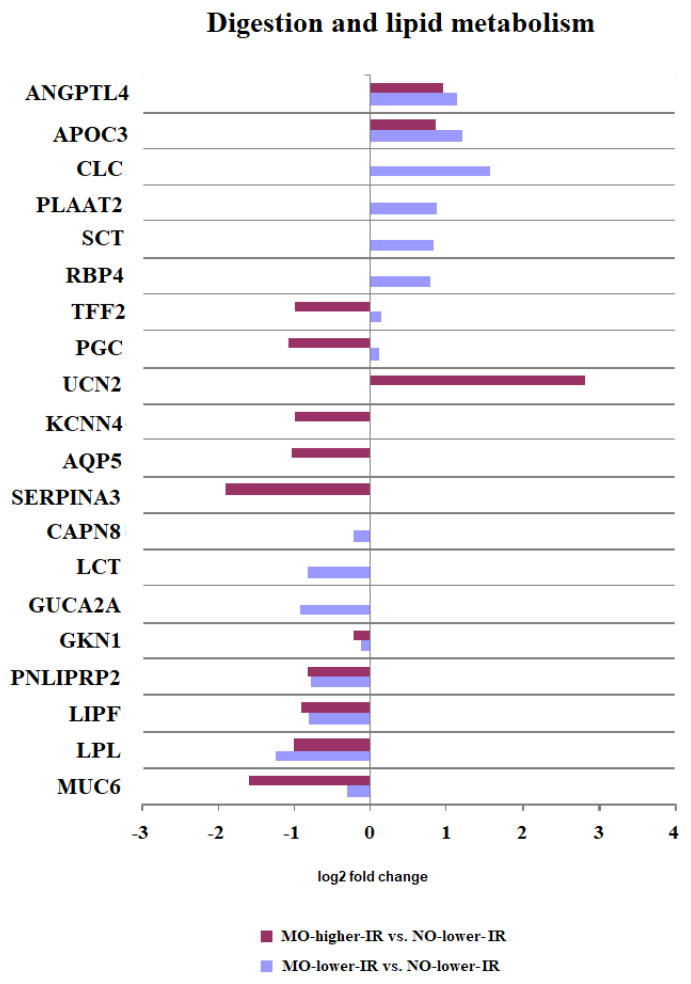
Representation of the Log_2_ of fold change of the differentially expressed genes (DEGs) included in the overrepresented gene sets (FDR < 0.05) related to digestion and lipid metabolism processes.

In the intersection of the two groups of DEGs, the overrepresented gene set (FDR < 0.05) showed an altered expression of genes in the duodenum that are related to digestion and lipid metabolism (Table 2 and Figure 2). There was a group of genes up- or downregulated in both MO groups regardless of the degree of insulin resistance. Lipases such as LPL, LIPF, and PNLIPRP2 were downregulated, while the inhibitors of lipase activity ANGPLT4 and APOC3 were upregulated in the duodenum of both MO groups. In addition, the mucin MUC6 was downregulated in both MO groups. Other genes, such as TFF2 and PGC were upregulated in MO-low-IR but downregulated in MO-high-IR (Table 2 and Figure 2).

However, there were genes involved in digestion that were expressed exclusively in each group of patients (Figure 2 and Table 2). For example, SCT and RBP4 were upregulated and LCT was downregulated in the MO-low-IR group.

#### 3.2.2. Defense Response and Inflammatory Processes

**Figure 3:**
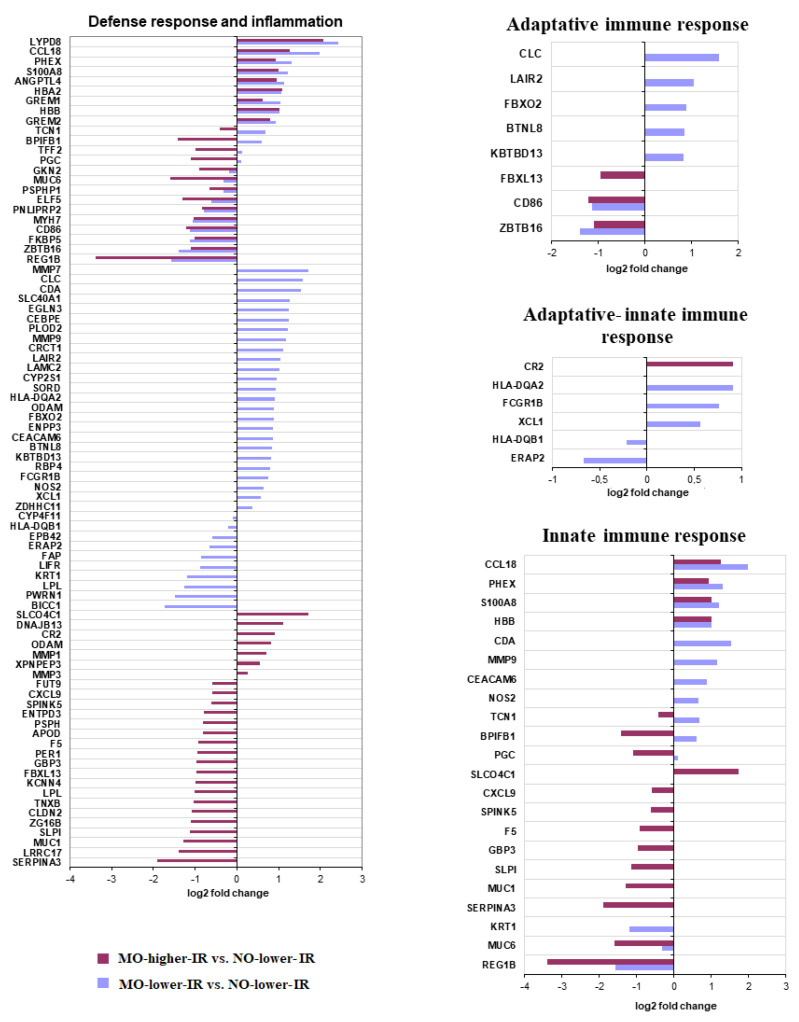
Representation of the Log_2_ of fold change of the differentially expressed genes (DEGs) included in the overrepresented gene sets (FDR < 0.05) related to defense response, inflammation, and innate and adaptive immune responses.

We found relevant results in the intersection of the two groups of DEGs and through the analysis of the genes involved in defensive functions and inflammation (Figure 3 and Table 2). According to the analysis with GSEA, many of these genes were related to gene sets of innate and adaptive immune responses (Supplementary Table S2).

We found shared upregulated and downregulated genes involved in both types of defensive responses regardless of the degree of insulin resistance. However, our results point to a trend towards increased expression of genes involved in adaptive and innate immune responses in MO-low-IR and a decrease in MO-high-IR (Table 2 and Figure 3).

**Figure 4:**
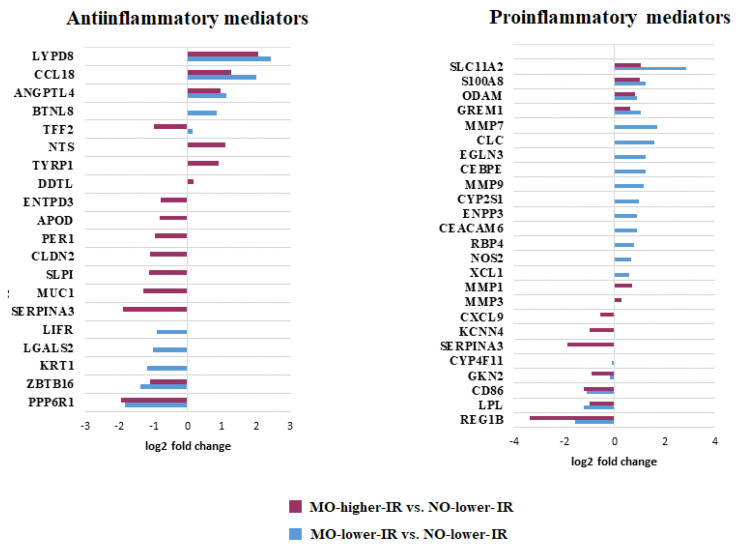
Representation of the Log_2_ of fold change of the differentially expressed genes (DEGs) related to pro- and anti-inflammatory processes.

Interestingly, according to the overrepresented gene set (FDR < 0.05) related to inflammatory processes and bibliographic analysis, both pro- and anti-inflammatory processes were altered in MO (Figure 4). Morbid obesity, regardless of the degree of insulin resistance, was associated with changes in the expression of genes involved in pro- (such as CD86 and S100A8) and anti-inflammatory (such as CCL18 and ZBTB16) responses (Figure 4). These genes are involved in different functions (Table 3). However, we found a different gene expression pattern depending on the insulin resistance level, mainly in pro-inflammatory responses. In the MO-low-IR group, there was a significant upregulation of genes associated with pro-inflammatory immune processes (such as XCL1, ENPP3, NOS2, CLC, CEBPE, RBP4, MMP9) (Figure 4 and Table 3). Additionally, we found a downregulation of certain genes related with pro-inflammatory processes (CXCL9, SERPINA3) in the MO-high-IR group (Figure 4).

**Figure 5:**
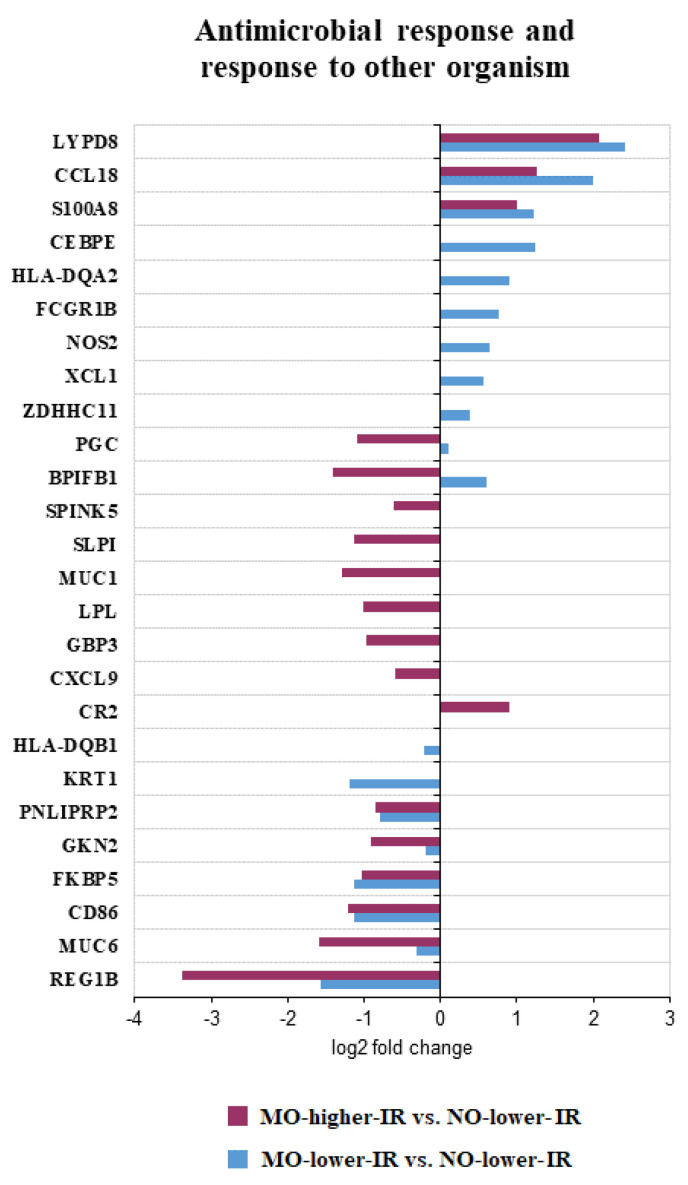
Representation of the Log_2_ of fold change of the differentially expressed genes (DEGs) included in the overrepresented gene sets (FDR < 0.05) related to antimicrobial response and response to another organism.

On the other hand, there was an overrepresentation (FDR < 0.05) of a gene set related to antimicrobial processes and responses to another organism (Supplementary Table S2). Our results indicate a defective response to external organisms in MO-high-IR, while in MO-low-IR it appears to be increased (Figure 5).

#### 3.2.3. Hypoxia

In the MO-low-IR group, there was an upregulation of genes involved in overrepresented (FDR < 0.05) gene sets related to hypoxia, although the hypoxia process does not seem to play a special role in MO-high-IR and in the intersection between the MO-low-IR and MO-high-IR groups (Table 2).

#### 3.2.4. Epithelial Maintenance, Wound Healing, and Homeostasis

In the intersection of the two groups of DEGs, we found changes in the expression of genes (FDR < 0.05) related to the maintenance of the gastrointestinal epithelium and epithelial structure (Table 2 and Supplementary Table S2). We found an upregulation of several genes in both MO groups regardless of the degree of insulin resistance (Table 2).

However, the functional analysis revealed mainly an upregulation of genes involved in wound healing functions and tissue homeostasis in the MO-low-IR group but not in the MO-high-IR group (Table 2).

#### 3.2.5. Cancer

In the analysis with GSEA, we found a significant overrepresentation (FDR < 0.05) of gene sets involved in the development of colorectal adenoma and gastric cancer in both MO groups (Table 2 and Supplementary Table S2). As shown in Supplementary Table S3, the changes in gene expression found in this study seem to be associated with the promotion of the migration, invasion, or proliferation of cancer cells.

## 4. Discussion

In this study, we analyzed changes in gene expression in the duodenum of two groups of women with morbid obesity, one with high and one with low insulin resistance, and compared them with a group of metabolically healthy women. First, we found a group of genes with a similar expression profile in both groups of women with morbid obesity compared to the control group; that is, genes upregulated and downregulated in morbid obesity regardless of the degree of insulin resistance. It is known that obesity is associated with a higher degree of systemic and local inflammation, where hypoxia and the release of cytokines and pro-inflammatory hormones are some of the factors that promote this process [39]. Thus, we have observed changes both in the expression of certain pro-inflammatory and anti-inflammatory genes, such as S100A8, GREM1, GREM2, and CCL18. S100A8 stimulates and promotes the migration of neutrophils and monocytes [40], with a critical role in intestinal pro-inflammatory responses [24,41]. In the same line, the increase in GREM1 and GREM2 expression could be associated with a hyperplasia of the duodenal crypts, which is characteristic of epithelial damage [42]. Nevertheless, we have also found an increase in the expression of the relevant anti-inflammatory chemokine CCL18, which is inducible by Th2 cytokines [43] and involved in M2 macrophage maturation [23], and a subexpression of CD86, which is associated with a pro-inflammatory state [44,45]. Thus, these results suggest the activation not only of pro-inflammatory responses in the duodenum in morbid obesity but also of synergistic anti-inflammatory mechanisms that could negatively regulate the possible collateral damage that occurs during inflammation [46].

We observed other DEGs that were related to lipid metabolism and digestion irrespective of the degree of insulin resistance. The downregulation of LPL, accompanied by the upregulation of two potent inhibitors of LPL enzyme, ANGPTL4 and APOC3 [47,48], was found. Increasing evidence suggests that LPL is regulated in a tissue-specific manner [49]. However, little is known at the intestinal level. In line with these results, we observed the downregulation of other lipases such as LIPF and PNLIPRP2. Overall, our data suggest deficient metabolism in the lipid degradation at the intestinal level in women with morbid obesity, which could constitute a protection mechanism in response to excessive fat intake but at the same time may contribute to the development of dyslipidemia, which could have a relevant role in the etiology of insulin resistance [50].

In morbid obesity, the microbiota could play a role as a regulator of metabolism [51]. Regardless of insulin resistance, the two groups of women with morbid obesity showed upregulation of LYPD8 and downregulation of a greater number of genes, such as REG1B, GKN2, LPL, PNLIPRP2, FKBP5, and CD86, that are related to responses to bacterial stimuli and antimicrobial activity. The microbiota could be involved in the change of CD86 expression since lipopolysaccharides (LPS) can alter its expression in both human peritoneal and M2 macrophages [52]. LYPD8 plays an important role in inhibiting the attachment of flagellated microbiota to colonic epithelia [53] and REG1B has antimicrobial effects [54]. On the other hand, the downregulation of LPL and PNLIPRP2 is not only involved in the regulation of lipid metabolism, but both also have antimicrobial effects. PNLIPRP2 displays its highest phospholipase activities on phosphatidylglycerol and phosphatidylethanolamine, two major constituents of bacterial membranes [55] with a supposed protective role against pathogenic microbiota [56,57]. In this sense, dietary lipids can exert pro- or anti-inflammatory functions on cells of the innate immune system and influence antigen presentation in cells of the adaptive immune system [58]. These results suggest a greater vulnerability of the intestine against microorganisms in patients with morbid obesity.

Nonetheless, our study has tried to delve deeper into the effects of high insulin resistance on the transcriptomic profile of the duodenum. Our results agree with a previous study where we observed an increase in the number of cytokines and chemokines in the duodenum of morbidly obese patients with low insulin resistance [15]. In the current study, we found a large number of upregulated genes involved in inflammatory processes related to the presence of various types of immune cells, such as M1 macrophages, T lymphocytes, NKT, eosinophils, basophils, and neutrophils, in women with morbid obesity and low insulin resistance. There was an increase in RBP4, which is expressed during the differentiation of monocytes into primary macrophages [59] and plays an important role in the development of insulin resistance in adipose tissue [60]. Moreover, an increase in NOS2 expression, which is induced in inflammatory processes by LPS [61] and hypoxia [62], was found. Its expression is characteristic of classically activated M1 macrophages in adipose tissue [31]. At the same time, we observed an increase in XCL1 expression, which is produced by T, NK, and NKT cells during infectious and inflammatory responses [63,64] and has bactericidal activity [65]. The expression of other genes is also increased, such as BTNL8, CLC, and PHD3 (Egln3). BTNL8 is highly expressed by neutrophils [34], which supports the idea that it has a regulatory role in inflammation [66]; along the same lines, other studies have shown the involvement of PHD3 in the hypoxic regulation of neutrophilic inflammation in humans and mice [67]. Moreover, the inhibition of PHD3 improves insulin sensitivity and ameliorates diabetes by specifically stabilizing HIF-2α [68]. On the other hand, CLC is typically expressed in eosinophils, and its increase is typically associated with altered epithelial barrier functions, including food-allergic enteropathies and inflammatory bowel diseases [69]. Although we found a large number of genes involved in pro-inflammatory responses, we have also observed the upregulation of S100G, a molecule that has been linked with anti-inflammatory effects [70]. However, the epithelial barrier function could be damaged by the observed upregulation of CEACAM6 and downregulation of LGALS2 in the MO-low-IR group. The increase in CEACAM6 in the intestinal epithelium has been linked with the massive colonization of adherent invasive *Escherichia coli* [71]. Additionally, LGALS2 confers maximum protection against exposure to pathogens [72].

It is known that obesity is associated with a greater degree of hypoxia in adipose and intestinal tissue, resulting in adverse metabolic effects like insulin resistance [73]. Hypoxia could contribute significantly to the changes found in the expression of genes in the duodenum of women with morbid obesity. In this sense, we have found an upregulation of different genes related to hypoxia regardless of the degree of insulin resistance, such as ANGPTL4, LPL, S100A8, SLC11A2, HBB, HBA2, and HBD. An increased expression and secretion of ANGPTL4 under hypoxic conditions has been observed in human adipocytes [74], and chronic intermittent hypoxia inhibits LPL by upregulating ANGPTL4 [75]. The activation of hypoxia signaling could induce the expression of the transporter SLC11A2, thus increasing the uptake of iron [76]. Additionally, an increase in the expression of S100A8 mediated by hypoxia has been observed in prostate cancer [77]. In addition, the upregulation of the three hemoglobins HBB, HBA2 and HBD may be indicative of a hypoxia situation [78]. However, there is another group of upregulated genes related to hypoxia in women with morbid obesity and low insulin resistance. This is in contrast to the literature that shows an association between hypoxia and insulin resistance [79]. However, part of these genes, in addition to participating in the regulation of hypoxia, could be involved in other different pathways, playing a beneficial role and supporting the maintenance of immune responses or tissue integrity and thereby influencing tissue recovery. For example, MMP7, which is induced by microbial products, can regulate tissue repair and plays an important role in the maintenance of innate immunity in the intestine, where it activates anti-bacterial peptides such as pro-defensins [80]. On the other hand, Egln3 (PHD3) not only functions as a tumor suppressor but may also promote fibrosis and anti-inflammatory responses and prevent neutrophil apoptosis under hypoxic conditions [81,82]. In addition, NOS2 produces NO, which is involved in the immune response as a defense mechanism; NO is responsible for inhibiting the production of IL-12 and macrophages. The possible functions of the rest of the genes related to hypoxic conditions are more unknown.

The downregulated genes found in the MO-high-IR group are mainly associated with defensive responses against bacteria (SPINK5, CXCL9, GBP3, SLPI, and MUC1). We have found a relation between both changes in microbiota and duodenal immune response in the presence of high insulin resistance in morbid obesity in our previous studies [15,83]. Moreover, we found that neurotensin expression was increased in women with high insulin resistance. The secretion of this molecule is stimulated by glucose [84] and inhibits the pro-inflammatory status of macrophages when administered under hyperglycemic conditions [85]. Together, our results suggest that there could be a relationship between the decrease in the duodenal immune response and the downregulation of genes involved in the maintenance of the gastrointestinal epithelium in the presence of high insulin resistance. This could constitute an underlying mechanism involved in the increase in the translocation of bacterial components, such as LPS, through the intestinal barrier in individuals with high insulin resistance [51].

Overweight and obesity are factors associated with an increased risk of cancer [86]. In this study, we found in the duodenum of women with morbid obesity a downregulation of a large number of genes that may be involved in antitumor processes, as well as the upregulation of genes with protumor activity in various types of cancer, mainly gastrointestinal cancers. For example, a lower expression of genes that may play an important role in suppressing gastric cancer (GKN1, LIPF, ANXA10, MUC6, PSCA, and SNHG5) was found. On the other hand, we have observed the upregulation of genes that are involved in the proliferation, migration, and invasion of cancer cells in colorectal and gastric cancer (ANGPTL4, S100A8, LINC00668). In the same way, we have obtained similar results in the exclusive DEGs of women with morbid obesity and high or low insulin resistance. Our results show the differential expression of many genes related with different types of cancer (see Supplementary Table S3) in the duodenum of women with morbid obesity, in whom there are still no signs of cancer development. However, we do not know if this altered gene expression will lead to the development of cancer in the future or if other factors are the main drivers of cancer development in this type of subject. Although small intestine cancer is not the most common form of gastrointestinal cancer, some studies point to an increased risk of developing it in patients with obesity [87,88]. However, due to the characteristics of this study, we do not know if the alteration in the expression of these genes may be related to an increased risk of developing small intestine cancer.

This study has several limitations. Firstly, this study was only carried out in women, so we cannot extrapolate the results to men. Additionally, although our results have shown an association between morbid obesity and insulin resistance with the expression of genes related to different pathways, e.g., cancer, more longitudinal studies are needed to confirm our findings.

## 5. Conclusions

In this study we have shown that morbid obesity, regardless of the degree of insulin resistance, is associated with alterations in intestinal defensive processes and antimicrobial responses and with the activation of pro- and anti-inflammatory genes, which could be associated with possible greater exposure and perhaps vulnerability of the intestine to products and metabolites derived from microorganisms and the diet. Similarly, we also found an alteration in pathways related to the lipid degradation at the intestinal level. More interestingly, we found that morbid obesity is associated with changes in the expression of certain genes that have been related to the development of cancer. Our data also suggest a deficient duodenal immune and antimicrobial response in women with high insulin resistance.

## Figures and Tables

**Table 1:** Anthropometric and biochemical variables of the three groups of women.

	NO-Lower-IR	MO-Lower-IR	MO-Higher-IR
N	6	7	8
Age (years)	43.6 (11.0)	48.0 (10.0)	45.0 (17.0)
Weight (kg)	55.5 (14.0)	110.6 (21.0) ^3^	126.3 (34.4) ^3^
BMI (kg/m^2^)	22.1 (6.9)	46.02 (6.7) ^3^	50.9 (14.3) ^3^
Waist (cm)	74.5 (14.3)	116.0 (18.0) ^3^	131.0 (10.5) ^3^
Hip (cm)	103.5 (6.0)	143 0 (31.0) ^2^	151.5 (23.5) ^2^
Glucose (mg/dL)	78.0 (13.0)	84.0 (7.0)	95.5 (19.0)
Insulin (µIU/mL)	8.0 (2.5)	10.5 (4.8)	24.3 (8.9) ^3,^*
Cholesterol (mg/dL)	194.5 (32.0)	186.0 (53.0)	188.0 (69.0)
Triglycerides (mg/dL)	83.5 (39.0)	110.0 (57.0)	142.0 (84.0)
HOMA-IR	1.47 (0.54)	2.13 (1.10)	5.2 (2.13) ^3,#^

Data given as median (interquartile range). Significant differences between MO-low-IR and MO-high-IR groups: * *p* < 0.05, ^#^ *p* < 0.01. Significant differences between NO-low-IR and MO-low-IR groups or between NO-low-IR and MO-high-IR groups: ^2^ *p* < 0.01, ^3^ *p* < 0.001. HOMA-IR, homeostatic model assessment of insulin resistance index; MO-high-IR, women with morbid obesity with high insulin resistance; MO-low-IR, women with morbid obesity with low insulin resistance; NO-low-IR, nonobese women with low insulin resistance.

**Table 2:** Significantly overrepresented (FDR < 0.05) gene sets involved in different functions obtained from the analysis of differentially expressed genes (DEGs) found in the intersection, from exclusive DEGs found in MO-low-IR and from exclusive DEGs found in MO-high-IR.

Function ^a^	Exclusive DEGs in MO-Lower-IR	DEGs in Intersection	Exclusive DEGs in MO-Higher-IR
Defense response and inflammation [1]	**BTNL8, CDA, CEACAM6, CEBPE, CLC, CRCT1, CYP2S1, EGLN3, ENPP3, FBXO2, FCGR1B, HLA-DQA2, KBTBD13, LAIR2, LAMC2, MMP7, MMP9, NOS2, PLOD2, RBP4, SLC40A1, SORD, XCL1, ZDHHC11, BICC1, CYP4F11**, EPB42, ERAP2, FAP, HLA-DQB1, KRT1, LIFR, PWRN1	ANGPTL4, BPIFB1 *, CCL18, GREM1, GREM2, HBA2, HBB, LYPD8, ODAM, PGC *, PHEX, S100A8, TFF2 *, TCN1 *, CD86, ELF5, FKBP5, GKN2, LPL, MUC6, MYH7, PNLIPRP2, PSPHP1, REG1B, ZBTB16	**CR2, DNAJB13, MMP1, MMP3, SLCO4C1, XPNPEP3,** APOD, CXCL9, ENTPD3, F5, FBXL13, FUT9, GBP3, KCNN4, LRRC17, MUC1, PCDHGC3, PER1, PSPH, SERPINA3, SERPINA5, SLPI, SPINK5, TNXB, ZG16B
Adaptative immune response [2]	**BTNL8, CLC, FBXO2, FCGR1B, HLA-DQA2, KBTBD13, LAIR2, XCL1,** ERAP2, HLA-DQB1	CD86, ZBTB16	**CR2,** FBXL13
Innate immune response [3]	**CDA, CEACAM6, FCGR1B, HLA-DQA2, MMP9, NOS2, XCL1,** ERAP2, HLA-DQB1, KRT1	BPIFB1 *, CCL18, HBB, PGC *, PHEX, S100A8, TCN1 *, MUC6, REG1B	**CR2,SLCO4C1,** CXCL9, F5, GBP3, MUC1, SERPINA3, SLPI, SPINK5
Inflammation [4]	**ENPP3,LAMC2, MMP9, NOS2, RBP4, XCL1,** BICC1, CYP4F11, EPB42, KRT1, HLA-DQB1	**CCL18, HBA2, HBB, ODAM,S100A8,** LPL, ZBTB16	**CR2, DNAJB13, MMP1, MMP3, XPNPEP3,** APOD, CXCL9, F5, PER1, PSPH, SERPINA3, SPINK5
Mediators in the production, signaling and response to cytokines [5]	**FCGR1B, HLA-DQA2, MMP9, NOS2, XCL1,** HLA-DQB1, LIFR	CCL18, GREM2, TFF2 *, CD86	**MMP1, MMP3,** CXCL9, GBP3, MUC1,
Antimicrobial response [6]	**CEBPE,FCGR1B, HLA-DQA2, NOS2, XCL1, ZDHHC11,** HLA-DQB1, KRT1	BPIFB1 *, CCL18, LYPD8, PGC *, S100A8, CD86, FKBP5, GKN2, LPL, MUC6, PNLIPRP2, REG1B	**CR2,** CXCL9, GBP3, MUC1, SLPI, SPINK5
Hypoxia [7]	**ARSL, EGLN3, MMP7, NOS2, PLOD2, SLC6A8, SLC6A10P**, CYP4F11, HLA-DQB1	**ANGPTL4**	APOD, MUC1
Epithelial maintenance and wound healing [8]	**CYP4F2, RBP4,** CYP4F11, FAP, KRT1	HBD, HBB, ODAM, S100A8, TFF2 *, MUC6	**HBG1,** APOD, F5, SERPINA3, SERPINA5
Homeostasis [9]	**CYP4F2, ERN1, NOS2, PM20D1, RBP4, SCT, SLC30A10, SLC40A1, XCL1,** EPB42, FTO, KRT1	APOC3, ANGPTL4, S100A8, SLC11A2, TFF2 *, CCDC66, CLRN1, HMBOX1, JPH4, LPL, MUC6	**MYOC, SCN3B,** CXCL9, KCNN4, SERPINA3, ZG16B
Fucosylation [10]	**FUT2**		FUT9, FUT1
Cell proliferation [11]	**CEACAM6, CLC, EGLN3, ENPP3, ERN1, FBXO2, FUT2, LAMC2, MMP9, NOS2, RBP4, SPEG, XCL1,** FTO, FAP, LIFR	**A4GNT, GREM1, ODAM,** CD86, GKN2, GKN1, REG1B, ZBTB16	APOD, CXCL9, FUT1, NCCRP1
Digestion and lipid metabolism [12]	**CLC, PLAAT2, RBP4, SCT,** CAPN8, GUCA2A, LCT	ANGPTL4, APOC3, PGC *, TFF2 *, GKN1, MUC6, LPL, LIPF, PNLIPRP2	**UCN2,** AQP5, KCNN4, SERPINA3
Response to biotic stimulus [13]	**CEBPE, FCGR1B, HLA-DQA2, NOS2, XCL1, ZDHHC11,** FAP, HLA-DQB1, KRT1	BPIFB1 *, CCL18, LYPD8, PGC *, S100A8, CD86, FKBP5, GKN2, LPL, MUC6, PNLIPRP2, REG1B	**CR2,** CXCL9, GBP3, MUC1, SLPI, SPINK5
Metabolic process [14]	**CDA, CYP4F2, CYP2S1, FUT2, LDHC, MMP7, MMP9, NOS2, PLAAT2, PLOD2, PM20D1, RBP4, SCT, SORD, SULT1C2, SLC6A8,** CYP4F11, FAP, UGT2B15	**APOC3, ANGPTL4, HBD, HBB, HBA2,** LPL, LIPF, PNLIPRP2, PSPHP1	**HBG1, MMP3, MMP1, TYRP1,** APOD, CYP2D6, ENTPD3, FADS6, FUT1, FUT9, PSPH, SERPINA3, TNXB
Transport [15]	**AQP12A, SCT, SLC30A10, CYP4F2, MMP9, NOS2, PM20D1, RBP4, SLC40A1, SLC6A8, XCL1,** KCNJ13	APOC3, GREM1, HBD, HBB, HBA2, S100A8, SLC11A2, TFF2 *, TCN1 *, JPH4, LPL, VPS18	**HBG1, KCNN2, KCNK9, SCN3B, SLCO4C1,** AQP5, APOD, CXCL9, GABRB3, KCNN4, KCNE2, PER1
Proteolysis [16]	**EGLN3, FBXO2, MMP9, MMP7, PM20D1, XPNPEP2,** ERAP2, FAP, KLK12, SPINK4	PHEX, PGC *, S100A8, C17orf97	**MMP1, MMP3, XPNPEP3,** CPO, FBXL13, KCNE2, NCCRP1, SLPI, SERPINA5, SPINK5, SERPINA3
Response to xenobiotics [17]	**CDA, CYP4F2, CYP2S1, RBP4,NOS2, SORD,** UGT2B15	LPL, NAT8	CYP2D6, KCNE2
Response to drug [18]	**CYP2S1, NOS2, SORD,** SPINK4	LPL, NAT8	APOD, CYP2D6, KCNE2
Response to toxic substance [19]		**HBA2, HBB, HBD**	**HBG1,** GSTT1
Gastrointestinal cancer [20]	**BTNL8, CDA, LAMC2, MMP7,PLAAT2, SULT1C2, SLC6A8, TMED6, XPNPEP2, ZDHHC11,** GAS5, GUCA2A, HLA-DQB1, HOXC6, LGALS2, LIFR, UGT2B15	APOC3, GREM2, LCN15, LYPD8, HBB, ODAM, PGC *, S100A8, TFF2 *, TCN1 *, C6orf58, FKBP5, GKN1, GKN2, LPL, LIPF, PNLIPRP2, REG1B, RERE, UCA1, ZBTB16	**CR2, FDCSP, MMP1, MMP3, MYOC, XPNPEP3, TYRP1,** CLDN2, C16orf89, ENTPD3, FUT9, GABRB3, HLF, KCNE2, KCNN4, LRRC17, MUC1, SERPINA3, SLPI, SPINK5, VSIG2

Upregulated DEGs are marked in bold. * DEGs in the intersection that are upregulated in MO-low-IR and downregulated in MO-high-IR relative to the control group are in bold and marked with an asterisk. ^a^ The numbers of these functions are described in Supplementary Table S2 and they include different significantly overrepresented (FDR < 0.05) gene sets.

**Table 3:** Bibliographic analysis of differentially expressed genes (DEGs) (FDR < 0.05) that are expressed or involved in the migration, proliferation, activation, or maturation of different immune cells.

	Direction of the Gene Expression in MO	DEG	Expression or Action in Immune Cells	Reference
DEGs from the intersection	Upregulated	CCL18	CCL18 causes maturation of cultured monocytes to macrophages in the M2 spectrum.	[23]
		S100A8	S100A8 induces mucosal CD4+ T cells with a Th1 pro-inflammatory response.	[24]
			S100A8/A9 is constitutively expressed in immune and epithelial cells of inflamed tissues.	[25]
	Downregulated	ZBTB16	ZBTB16 controls the development of invariant natural killer T cell effector functions.	[26]
		CD86	CD86 is typically found on the surface of antigen-presenting cells and can either bind CD28 or CTLA-4, resulting in a costimulatory or a co-inhibitory response, respectively.	[27]
Exclusive DEGs from MO-low-IR	Upregulated	XCL1	XCL1 is produced mainly by NK and activated CD8+ T cells and facilitates the activation and migration of intestinal dendritic cells.	[28]
		ENPP3	ENPP3 prevents a decrease in plasmacytoid dendritic cell numbers in the small intestine.	[29]
			ENPP3 is highly expressed in activated basophils and mast cells and is rapidly induced by IgE.	[30]
		NOS2	An increase in Nos2 expression is characteristic of classically activated M1 macrophages.	[31]
		CLC	CLC has lysophospholipase activity and is a characteristic constituent of eosinophils and basophils.	[32]
		CEBPE	CEBPE is an essential transcription factor for granulocytic differentiation.	[33]
		BTNL8	BTNL8 is highly expressed on neutrophils.	[34]
		RBP4	RBP4 induces antigen-presenting cells as the drivers of an inflammatory response.	[35]
		MMP9	MMP9 recruits neutrophils to sites of inflammation.	[36]
	Downregulated	HLA-DQB1	HLA-DQB1 is expressed in antigen presenting cells.	[37]
Exclusive DEGs from MO-high-IR	Downregulated	CXCL9	CXCL9 is secreted in response to IFN-γ. Mainly secreted by monocytes, endothelial cells, fibroblasts, and cancer cells in response to IFN-γ	[38]

## Data Availability

The data discussed in this publication have been deposited in the NCBI’s Gene Expression Omnibus (GEO) (GEO Series access number GSE147562).

## Supplementary Materials

The following supporting information can be downloaded at: https://www.mdpi.com/article/10.3390/biomedicines10051024/s1, Supplementary Table S1. Anthropometric and biochemical variables for each patient of the three groups of women; Supplementary Table S2. Complete analysis of the overrepresented gene sets (FDR < 0.05), the associated *p*-value and FDR *q*-value, and the differentially expressed genes (DEGs); Supplementary Table S3. Bibliographical analysis of differentially expressed genes (DEGs) (FDR < 0.05) involved in the development of cancer [89,90,91,92,93,94,95,96,97,98,99,100,101,102,103,104,105,106,107,108,109,110,111,112,113,114,115,116,117,118,119,120,121,122,123,124,125,126,127,128,129,130,131,132,133].
