# Supplementary material for: Morbid Obesity in Women Is Associated with an Altered Intestinal Expression of Genes Related to Cancer Risk and Immune, Defensive, and Antimicrobial Response
Source: Biomedicines. 2022 Apr 29;10(5):1024. doi: 10.3390/biomedicines10051024 (PMC9138355; doi:10.3390/biomedicines10051024)
Supplement: Supplementary file 1 [file biomedicines-10-01024-s001.zip › Supplementary Table S1 R1.pdf]

Supplementary Table S1. -Anthropometric and biochemical variables for each patient of the three groups of women.

		Age (years)	Weight (kg)	BMI (Kg/m^2)	Waist (cm)	Hip (cm)	Glucose (mg/dl)	Insulin (µIU/ml)	Cholesterol (mg/dl)	Triglycerides (mg/dl)	HOMA-IR
**NO-lower-IR**	Patient 1	53	63	25.2	89	105	78	8.2	210	68	1.59
	Patient 2	40	70	27.3	82	107	78	13.0	190	75	2.50
	Patient 3	38	51	17.6	75	95	68	7.7	173	149	1.30
	Patient 4	47	58	22.7	70	105	77	8.3	162	95	1.58
	Patient 5	47	53	21.5	68	101	78	7.1	199	70	1.38
	Patient 6	37	50	19.3	74	102	81	6.1	199	92	1.23
**MO-lower-IR**	Patient 1	52	128	46.5	136	160	87	14.0	160	110	3.01
	Patient 2	49	108	43.8	116	130	80	10.0	213	135	1.99
	Patient 3	42	110	46.0	113	143	85	8.4	209	114	1.78
	Patient 4	44	137	55.0	125	160	72	12.0	151	80	2.14
	Patient 5	56	123	50.5	130	147	84	9.1	186	58	1.91
	Patient 6	48	96	40.0	107	129	83	10.5	178	78	2.15
	Patient 7	37	107	43.9	112	125	87	17.0	215	185	3.66
**MO-higher-IR**	Patient 1	32	129	49.8	129	153	98	29.7	180	156	7.19
	Patient 2	61	119	41.9	136	143	101	18.3	154	146	4.58
	Patient 3	53	105	43.9	116	131	103	19.4	196	103	4.95
	Patient 4	45	148	57.1	130	165	93	24.6	213	210	5.65
	Patient 5	44	150	57.5	144	160	89	24.1	169	202	5.30
	Patient 6	49	161	55.7	141	159	104	50.2	231	138	12.90
	Patient 7	45	123	52.2	132	150.	82	25.2	247	117	5.10
	Patient 8	31	114	42.0	130	134	82	20.4	153	47	4.13
