# Supplementary material for: Morbid Obesity in Women Is Associated with an Altered Intestinal Expression of Genes Related to Cancer Risk and Immune, Defensive, and Antimicrobial Response
Source: Biomedicines. 2022 Apr 29;10(5):1024. doi: 10.3390/biomedicines10051024 (PMC9138355; doi:10.3390/biomedicines10051024)
Supplement: Supplementary file 1 [file biomedicines-10-01024-s001.zip › Supplementary table S3.pdf]

Supplementary table S3. Bibliographical analysis of differentially expressed genes (DEGs) (FDR<0.05) involved in the development of cancer.

	Direction of the gene expression in MO	DEGs	Potential activity	Contribution to the development of cancer according bibliography	Subjects or samples of the study	References
**DEGs from the intersection**	Upregulated	ANGPTL4	Protumoral	Upregulation of ANGPTL4 in 50% of human colorectal cancers.	- Human samples	[1]
				The upregulation of ANGPTL4 promoted cell proliferation and tumor growth.	- Human cell lines<br>- Mice	[1]
		S100A8	Protumoral	S100A8 promoted invasion and migration of gastric cancer cells.	- Human cell lines	[2]
		LINC00668	Protumoral	Upregulation of LINC00668 in human gastric cancer tissues, and it correlated with poor prognosis.	- Human samples	[3]
				LINC00668 promoted gastric cancer cell proliferation.	- Human cell lines<br>- Mice	[3]
	Downregulated	ZBTB16	Tumor suppressor	The downregulation of PLZF was involved in the tumorigenesis of various types of cancers.	- Human samples<br>- Human cell lines	[4]
		CD86	Tumor suppressor	The upregulation of CD86 increased the ability of NK cells to kill tumor cells.	- Mice	[5]
		GKN1	Tumor suppressor	The upregulation of GKN1 induced gastric cancer cells apoptosis.<br>Downregulation of GKN1 during gastric cancer progression.	- Human samples<br>- Human cell lines	[6]
				GKN1 inhibited gastric cancer cell migration and invasion.	- Human samples	[7]
		GKN2	Tumor suppressor	The loss of GKN2 drove premalignant gastric inflammation and tumor progression.	- Mice	[8]
		LIPF	Tumor suppressor	The downregulation of LIPF predicted poor outcome of gastric cancer.	- Human samples	[9]
		ANXA10	Tumor suppressor	Downregulation of annexin A10 in gastric cancer.<br>Upregulation in tumor cell growth suppression.	- Human samples<br>- Human cell lines	[10]
		MUC6	Tumor suppressor	MUC6 expression inhibited tumor cell invasion.	- Human samples<br>- Human and animal cell lines	[11]
		PSCA	Tumor suppressor	PSCA expression was associated with favourable tumor features.<br>PSCA reduced PSA recurrence in operated prostate cancer.	- Human samples	[12]
		SNHG5	Tumor suppressor	Downregulation of SNHG5 in gastric cancer.<br>SNHG5 suppressed gastric cancer progression.	- Human samples<br>- Human cell lines	[13]
		MSMB	Tumor suppressor	Tumor suppressor	- Human samples	[14, 15, 16]
		UCA1	Protumoral	The upregulation of UCA1 in colorectal cancer influenced cell proliferation, apoptosis and cell cycle distribution.	- Human samples<br>- Human cell lines	[17]
**Exclusive DEGs from MO-lower-IR**	Upregulated	EGLN3	Tumor suppressor	EGLN3/PHD3 may act as a tumor suppressor in gastric cancer.	- Human samples<br>- Human cell lines	[18]
		NOS2	Protumoral	Nos2 may play an important role in the development of intestinal tumors.	- Mice	[19]
		CEBPE	Protumoral	C/EBPβ was upregulated in colorectal carcinoma cell cultures.	- Human samples	[20]

				C/EBPβ upregulation promoted tumor cell invasion *in vitro* and was associated with metastatic status in colorectal cancer.	- Human cell line	
		LDHC	Protumoral	LDHC was upregulated in renal cell carcinoma tissues and associated with poor prognosis in renal cell carcinoma.<br>LDHC played a pivotal role in the migration and invasion of renal cell carcinoma cells.	- Human samples<br>- Human cell lines	[21]
		LAMC2	Protumoral	The upregulation of LAMC2 on cancer cells appeared to drive tumorigenesis.<br>The upregulation of LAMC2 in cancer patients might be a serum-based diagnostic biomarker.	- Human samples<br>- Human cell lines	[22]
		MMP9	Protumoral	MMP9 could be a critical molecule for invasion and metastasis in renal cell carcinoma and was closely associated with poor survival.	- Human samples	[23]
		PLOD2	Protumoral	PLOD2 induced under hypoxia was a novel prognostic factor for hepatocellular carcinoma after curative resection.	- Human samples<br>- Human cell lines	[24]
				PLOD2 promoted aerobic glycolysis and cell progression in colorectal cancer.	- Human samples<br>- Human cell lines	[25]
		SLC6A10P	Protumoral	SLC6A10P was highly expressed in tumor tissues and could predict poor survival in patients with lung adenocarcinoma.	-Human samples	[26]
		MMP7	Protumoral	MMP7 was upregulated by tumor-associated macrophages in hypoxic areas of human tumors.	-Human samples<br>-Human cell cultures	[27]
		CRCT1	Tumor suppressor	CRCT1 may act as a tumor suppressor gene in esophageal squamous cell carcinoma.	- Human cell lines<br>- Human samples	[28]
		PIWIL2	Protumoral	PIWIL2 expression was relatively higher in colorectal carcinoma.	- Human samples	[29]
		CEACAM6	Protumoral	CEACAM6 expression was elevated in many solid tumors.<br>CEACAM6 has a role in tumor cell migration, invasion and adhesion and formation of distant metastases.	- Human samples<br>- Human cell lines	[30]
		LIFR	Tumor suppressor	LIFR was a breast cancer metastasis suppressor and a prognostic marker.	- Human and animal cell lines<br>- Human samples<br>- Mice	[31]
				A reduced expression of LIFR was found in many cancers.	- Human samples<br>- Human cell lines	[32]
		KLK12	Protumoral	The KLK12 gene was markedly overexpressed in gastric cancer tissue.	-Human samples	[33]
		GAS5	Tumor suppressor	The downregulation of long noncoding RNA GAS5 indicated a poor prognosis and promotes cell proliferation in gastric cancer.	- Human cell lines<br>- Human samples	[34]
		PWRN1	Tumor suppressor	PWRN1 suppressed gastric cancer growth as a competing endogenous RNA of microRNA-425-5p.	-Human samples<br>-Human cell lines	[35]
**Exclusive DEGs from MO-higher-IR**	Upregulated	MMP1	Protumoral	Upregulation of MMP1 was involved in gastric cancer cell invasion.	- Human cell lines	[36]
		MMP3	Protumoral	MMP3 was upregulated by C/EBPβ overexpression in HCT116 colorectal cancer cell cultures.	- Human cell line<br>- Human samples	[20]

				C/EBPβ upregulation promoted tumor cell invasion in an MMP3-dependent manner *in vitro* and was associated with metastatic status in colorectal cancer.		
		KCNK9	Protumoral	The upregulation of KCNK9 may contribute to the development of colorectal cancers promoting tumor formation and inducing resistance to both hypoxia and serum deprivation.	- Human samples	[37]
		TNXB	Tumor suppressor	TNX expression was significantly downregulated during cancer progression.<br>TNXB expression in cancer was correlated with a good survival prognosis.	- Human samples	[38]
		FUT9	Tumor suppressor	FUT9 downregulation enhanced the invasive behavior of bulk colon cancer cells.	- Human cell lines<br>- Mice	[39]
		SERPINA5	Tumor suppressor	SERPINA5 inhibited tumor cell migration in hepatocellular carcinoma.	- Human cell lines<br>- Human samples	[40]
				Loss of SERPINA5 protein expression was associated with advanced-stage serous ovarian tumors.	- Human samples	[41]
		PER1	Tumor suppressor	The circadian clock gene PER1 suppressed cancer cell proliferation and tumor growth at specific times of day.	- Murine cell line<br>- Mice	[42]
				Correlated downregulation of estrogen receptor beta and the circadian clock gene Per1 in human colorectal cancer.	-Human samples	[43]
		PSPH	Protumoral	Increased PSPH levels was observed in a number of human tumor types including non-small-cell lung cancer.	- Human samples<br>- Mouse samples	[44]
		MUC1	Protumoral	MUC1 gene silencing may represent a novel therapeutic approach in the treatment of a variety of human cancers.	- Human cell line	[45]

REFERENCES

1. Kim SH, Park YY, Kim SW, Lee JS, Wang D, DuBois RN. ANGPTL4 induction by prostaglandin E2 under hypoxic conditions promotes colorectal cancer progression. Cancer Res. 2011 Nov 15;71(22):7010-20. doi: 10.1158/0008-5472.CAN-11-1262. Epub 2011 Sep 21. PMID: 21937683; PMCID: PMC3217078.
2. Kwon CH, Moon HJ, Park HJ, Choi JH, Park DY. S100A8 and S100A9 promotes invasion and migration through p38 mitogen-activated protein kinase-dependent NF-κB activation in gastric cancer cells. Mol Cells. 2013 Mar;35(3):226-34. doi: 10.1007/s10059-013-2269-x. Epub 2013 Feb 26. PMID: 23456298; PMCID: PMC3887919.
3. Dianatpour A, Ghafouri-Fard S. Long Non Coding RNA Expression Intersecting Cancer and Spermatogenesis: A Systematic Review. Asian Pac J Cancer Prev. 2017 Oct 26;18(10):2601-2610. doi: 10.22034/APJCP.2017.18.10.2601. PMID: 29072050; PMCID: PMC5747377.
4. Hui AW, Lau HW, Cao CY, Zhou JW, Lai PB, Tsui SK. Downregulation of PLZF in human hepatocellular carcinoma and its clinical significance. Oncol Rep. 2015 Jan;33(1):397-402. doi: 10.3892/or.2014.3578. Epub 2014 Oct 30. PMID: 25369784.
5. Peng Y, Luo G, Zhou J, Wang X, Hu J, Cui Y, Li XC, Tan J, Yang S, Zhan R, Yang J, He W, Wu J. CD86 is an activation receptor for NK cell cytotoxicity against tumor cells. PLoS One. 2013 Dec 11;8(12):e83913. doi: 10.1371/journal.pone.0083913. PMID: 24349559; PMCID: PMC3859666.

6. Mao W, Chen J, Peng TL, Yin XF, Chen LZ, Chen MH. Downregulation of gastrokine-1 in gastric cancer tissues and restoration of its expression induced gastric cancer cells to apoptosis. J Exp Clin Cancer Res. 2012 May 23;31(1):49. doi: 10.1186/1756-9966-31-49. PMID: 22621392; PMCID: PMC3511871.
7. Yoon JH, Choi WS, Kim O, Choi BJ, Nam SW, Lee JY, Park WS. Gastrokine 1 inhibits gastric cancer cell migration and invasion by downregulating RhoA expression. Gastric Cancer. 2017 Mar;20(2):274-285. doi: 10.1007/s10120-016-0617-1. Epub 2016 Jun 1. PMID: 27250838.
8. Menheniott TR, O'Connor L, Chionh YT, Däbritz J, Scurr M, Rollo BN, Ng GZ, Jacobs S, Catubig A, Kurklu B, Mercer S, Minamoto T, Ong DE, Ferrero RL, Fox JG, Wang TC, Sutton P, Judd LM, Giraud AS. Loss of gastrokine-2 drives premalignant gastric inflammation and tumor progression. J Clin Invest. 2016 Apr 1;126(4):1383-400. doi: 10.1172/JCI82655. Epub 2016 Mar 14. PMID: 26974160; PMCID: PMC4811116.
9. Kong Y, Zheng Y, Jia Y, Li P, Wang Y. Decreased LIPF expression is correlated with DGKA and predicts poor outcome of gastric cancer. Oncol Rep. 2016 Oct;36(4):1852-60. doi: 10.3892/or.2016.4989. Epub 2016 Aug 1. PMID: 27498782; PMCID: PMC5022960.
10. Kim JK, Kim PJ, Jung KH, Noh JH, Eun JW, Bae HJ, Xie HJ, Shan JM, Ping WY, Park WS, Lee JY, Nam SW. Decreased expression of annexin A10 in gastric cancer and its overexpression in tumor cell growth suppression. Oncol Rep. 2010 Sep;24(3):607-12. PMID: 20664964.
11. Leir SH, Harris A. MUC6 mucin expression inhibits tumor cell invasion. Exp Cell Res. 2011 Oct 15;317(17):2408-19. doi: 10.1016/j.yexcr.2011.07.021. Epub 2011 Aug 5. PMID: 21851820.
12. Heinrich MC, Göbel C, Kluth M, Bernreuther C, Sauer C, Schroeder C, Möller-Koop C, Hube-Magg C, Lebok P, Burandt E, Sauter G, Simon R, Huland H, Graefen M, Heinzer H, Schlomm T, Heumann A. PSCA expression is associated with favorable tumor features and reduced PSA recurrence in operated prostate cancer. BMC Cancer. 2018 May 31;18(1):612. doi: 10.1186/s12885-018-4547-7. PMID: 29855276; PMCID: PMC5984312.
13. Zhao L, Guo H, Zhou B, Feng J, Li Y, Han T, Liu L, Li L, Zhang S, Liu Y, Shi J, Zheng D. Long non-coding RNA SNHG5 suppresses gastric cancer progression by trapping MTA2 in the cytosol. Oncogene. 2016 Nov 3;35(44):5770-5780. doi: 10.1038/onc.2016.110. Epub 2016 Apr 11. PMID: 27065326.
14. Bergström SH, Järemo H, Nilsson M, Adamo HH, Bergh A. Prostate tumors downregulate microseminoprotein-beta (MSMB) in the surrounding benign prostate epithelium and this response is associated with tumor aggressiveness. Prostate. 2018 Mar;78(4):257-265. doi: 10.1002/pros.23466. Epub 2017 Dec 18. PMID: 29250809.
15. Dahlman A, Rexhepaj E, Brennan DJ, Gallagher WM, Gaber A, Lindgren A, Jirström K, Bjartell A. Evaluation of the prognostic significance of MSMB and CRISP3 in prostate cancer using automated image analysis. Mod Pathol. 2011 May;24(5):708-19. doi: 10.1038/modpathol.2010.238. Epub 2011 Jan 14. PMID: 21240253.
16. Whitaker HC, Kote-Jarai Z, Ross-Adams H, Warren AY, Burge J, George A, Bancroft E, Jhavar S, Leongamornlert D, Tymrakiewicz M, Saunders E, Page E, Mitra A, Mitchell G, Lindeman GJ, Evans DG, Blanco I, Mercer C, Rubinstein WS, Clowes V, Douglas F, Hodgson S, Walker L, Donaldson A, Izatt L, Dorkins H, Male A, Tucker K, Stapleton A, Lam J, Kirk J, Lilja H, Easton D; IMPACT Study Steering Committee; IMPACT Study Collaborators; UK GPCS Collaborators, Cooper C, Eeles R, Neal DE. The rs10993994 risk allele for prostate cancer results in clinically relevant changes in microseminoprotein-beta expression in tissue and urine. PLoS One. 2010 Oct 13;5(10):e13363. doi: 10.1371/journal.pone.0013363. PMID: 20967219; PMCID: PMC2954177.
17. Han Y, Yang YN, Yuan HH, Zhang TT, Sui H, Wei XL, Liu L, Huang P, Zhang WJ, Bai YX. UCA1, a long non-coding RNA up-regulated in colorectal cancer influences cell proliferation, apoptosis and cell cycle distribution. Pathology. 2014 Aug;46(5):396-401. doi: 10.1097/PAT.0000000000000125. PMID: 24977734.
18. Xia YJ, Jiang XT, Jiang SB, He XJ, Luo JG, Liu ZC, Wang L, Tao HQ, Chen JZ. PHD3 affects gastric cancer progression by negatively regulating HIF1A. Mol Med Rep. 2017 Nov;16(5):6882-6889. doi: 10.3892/mmr.2017.7455. Epub 2017 Sep 11. PMID: 28901473.

19. Yerushalmi HF, Besselsen DG, Ignatenko NA, Blohm-Mangone KA, Padilla-Torres JL, Stringer DE, Cui H, Holubec H, Payne CM, Gerner EW. The role of NO synthases in arginine-dependent small intestinal and colonic carcinogenesis. Mol Carcinog. 2006 Feb;45(2):93-105. doi: 10.1002/mc.20168. PMID: 16329147.
20. Ji Y, Li J, Li P, Wang L, Yang H, Jiang G. C/EBPβ Promotion of MMP3-Dependent Tumor Cell Invasion and Association with Metastasis in Colorectal Cancer. Genet Test Mol Biomarkers. 2018 Jan;22(1):5-10. doi: 10.1089/gtmb.2017.0113. Epub 2017 Nov 27. PMID: 29172775.
21. Hua Y, Liang C, Zhu J, Miao C, Yu Y, Xu A, Zhang J, Li P, Li S, Bao M, Yang J, Qin C, Wang Z. Expression of lactate dehydrogenase C correlates with poor prognosis in renal cell carcinoma. Tumour Biol. 2017 Mar;39(3):1010428317695968. doi: 10.1177/1010428317695968. PMID: 28351304.
22. Garg M, Braunstein G, Koeffler HP. LAMC2 as a therapeutic target for cancers. Expert Opin Ther Targets. 2014 Sep;18(9):979-82. doi: 10.1517/14728222.2014.934814. Epub 2014 Jun 30. PMID: 24976367.
23. Cho NH, Shim HS, Rha SY, Kang SH, Hong SH, Choi YD, Hong SJ, Cho SH. Increased expression of matrix metalloproteinase 9 correlates with poor prognostic variables in renal cell carcinoma. Eur Urol. 2003 Nov;44(5):560-6. doi: 10.1016/s0302-2838(03)00362-2. PMID: 14572755.
24. Noda T, Yamamoto H, Takemasa I, Yamada D, Uemura M, Wada H, Kobayashi S, Marubashi S, Eguchi H, Tanemura M, Umeshita K, Doki Y, Mori M, Nagano H. PLOD2 induced under hypoxia is a novel prognostic factor for hepatocellular carcinoma after curative resection. Liver Int. 2012 Jan;32(1):110-8. doi: 10.1111/j.1478-3231.2011.02619.x. Epub 2011 Aug 11. PMID: 22098155.
25. Du W, Liu N, Zhang Y, Liu X, Yang Y, Chen W, He Y. PLOD2 promotes aerobic glycolysis and cell progression in colorectal cancer by upregulating HK2. Biochem Cell Biol. 2020 Jun;98(3):386-395. doi: 10.1139/bcb-2019-0256. Epub 2019 Nov 19. PMID: 31742425.
26. Yuan K, Gao ZJ, Yuan WD, Yuan JQ, Wang Y. High expression of SLC6A10P contributes to poor prognosis in lung adenocarcinoma. Int J Clin Exp Pathol. 2018 Feb 1;11(2):720-726. PMID: 31938158; PMCID: PMC6958030.
27. Lewis C, Murdoch C. Macrophage responses to hypoxia: implications for tumor progression and anti-cancer therapies. Am J Pathol. 2005 Sep;167(3):627-35. doi: 10.1016/S0002-9440(10)62038-X. PMID: 16127144; PMCID: PMC1698733.
28. Wu N, Song Y, Pang L, Chen Z. CRCT1 regulated by microRNA-520 g inhibits proliferation and induces apoptosis in esophageal squamous cell cancer. Tumour Biol. 2016 Jun;37(6):8271-9. doi: 10.1007/s13277-015-4730-2. Epub 2015 Dec 30. PMID: 26718216.
29. Oh SJ, Kim SM, Kim YO, Chang HK. Clinicopathologic Implications of PIWIL2 Expression in Colorectal Cancer. Korean J Pathol. 2012 Aug;46(4):318-23. doi: 10.4132/KoreanJPathol.2012.46.4.318. Epub 2012 Aug 23. PMID: 23110023; PMCID: PMC3479824.
30. Blumenthal RD, Leon E, Hansen HJ, Goldenberg DM. Expression patterns of CEACAM5 and CEACAM6 in primary and metastatic cancers. BMC Cancer. 2007 Jan 3;7:2. doi: 10.1186/1471-2407-7-2. PMID: 17201906; PMCID: PMC1769503.
31. Chen D, Sun Y, Wei Y, Zhang P, Rezaeian AH, Teruya-Feldstein J, Gupta S, Liang H, Lin HK, Hung MC, Ma L. LIFR is a breast cancer metastasis suppressor upstream of the Hippo-YAP pathway and a prognostic marker. Nat Med. 2012 Oct;18(10):1511-7. doi: 10.1038/nm.2940. Epub 2012 Sep 23. PMID: 23001183; PMCID: PMC3684419.
32. Zhao JH, Sun JX, Song YX, Chen XW, Yang YC, Ma B, Wang J, Gao P, Wang ZN. A novel long noncoding RNA-LOWEG is low expressed in gastric cancer and acts as a tumor suppressor by inhibiting cell invasion. J Cancer Res Clin Oncol. 2016 Mar;142(3):601-9. doi: 10.1007/s00432-015-2071-6. Epub 2015 Nov 4. PMID: 26537802.
33. Zhao EH, Shen ZY, Liu H, Jin X, Cao H. Clinical significance of human kallikrein 12 gene expression in gastric cancer. World J Gastroenterol. 2012 Dec 7;18(45):6597-604. doi: 10.3748/wjg.v18.i45.6597. PMID: 23236234; PMCID: PMC3516218.
34. Sun M, Jin FY, Xia R, Kong R, Li JH, Xu TP, Liu YW, Zhang EB, Liu XH, De W. Decreased expression of long noncoding RNA GAS5 indicates a poor prognosis and promotes cell proliferation in gastric cancer. BMC Cancer. 2014 May 6;14:319. doi: 10.1186/1471-2407-14-319. PMID: 24884417; PMCID: PMC4022532.

35. Chen Z, Ju H, Yu S, Zhao T, Jing X, Li P, Jia J, Li N, Tan B, Li Y. Prader-Willi region non-protein coding RNA 1 suppressed gastric cancer growth as a competing endogenous RNA of miR-425-5p. Clin Sci (Lond). 2018 May 23;132(9):1003-1019. doi: 10.1042/CS20171588. PMID: 29535266.
36. Bao W, Fu HJ, Jia LT, Zhang Y, Li W, Jin BQ, Yao LB, Chen SY, Yang AG. HER2-mediated upregulation of MMP-1 is involved in gastric cancer cell invasion. Arch Biochem Biophys. 2010 Jul;499(1-2):49-55. doi: 10.1016/j.abb.2010.05.009. Epub 2010 May 10. PMID: 20460098.
37. Kim CJ, Cho YG, Jeong SW, Kim YS, Kim SY, Nam SW, Lee SH, Yoo NJ, Lee JY, Park WS. Altered expression of KCNK9 in colorectal cancers. APMIS. 2004 Sep;112(9):588-94. doi: 10.1111/j.1600-0463.2004.apm1120905.x. PMID: 15601307.
38. Liot S, Aubert A, Hervieu V, Kholti NE, Schalkwijk J, Verrier B, Valcourt U, Lambert E. Loss of Tenascin-X expression during tumor progression: A new pan-cancer marker. Matrix Biol Plus. 2020 Jan 20;6-7:100021. doi: 10.1016/j.mbplus.2020.100021. PMID: 33543019; PMCID: PMC7852205.
39. Auslander N, Cunningham CE, Toosi BM, McEwen EJ, Yizhak K, Vizeacoumar FS, Parameswaran S, Gonen N, Freywald T, Bhanumathy KK, Freywald A, Vizeacoumar FJ, Ruppin E. An integrated computational and experimental study uncovers FUT9 as a metabolic driver of colorectal cancer. Mol Syst Biol. 2017 Dec 1;13(12):956. doi: 10.15252/msb.20177739. PMID: 29196508; PMCID: PMC5740504.
40. Jing Y, Jia D, Wong CM, Oi-Lin Ng I, Zhang Z, Liu L, Wang Q, Zhao F, Li J, Yao M, Wu X, He X. SERPINA5 inhibits tumor cell migration by modulating the fibronectin-integrin β1 signaling pathway in hepatocellular carcinoma. Mol Oncol. 2014 Mar;8(2):366-77. doi: 10.1016/j.molonc.2013.12.003. Epub 2013 Dec 18. PMID: 24388360; PMCID: PMC5528558.
41. Bijsmans IT, Smits KM, de Graeff P, Wisman GB, van der Zee AG, Slangen BF, de Bruïne AP, van Engeland M, Sieben NL, Van de Vijver KK. Loss of SerpinA5 protein expression is associated with advanced-stage serous ovarian tumors. Mod Pathol. 2011 Mar;24(3):463-70. doi: 10.1038/modpathol.2010.214. Epub 2010 Nov 19. PMID: 21102419.
42. Yang X, Wood PA, Ansell CM, Quiton DF, Oh EY, Du-Quiton J, Hrushesky WJ. The circadian clock gene Per1 suppresses cancer cell proliferation and tumor growth at specific times of day. Chronobiol Int. 2009 Oct;26(7):1323-39. doi: 10.3109/07420520903431301. PMID: 19916834.
43. Mostafaie N, Kállay E, Sauerzapf E, Bonner E, Kriwanek S, Cross HS, Huber KR, Krugluger W. Correlated downregulation of estrogen receptor beta and the circadian clock gene Per1 in human colorectal cancer. Mol Carcinog. 2009 Jul;48(7):642-7. doi: 10.1002/mc.20510. PMID: 19148895.
44. Bachelor MA, Lu Y, Owens DM. L-3-Phosphoserine phosphatase (PSPH) regulates cutaneous squamous cell carcinoma proliferation independent of L-serine biosynthesis. J Dermatol Sci. 2011 Sep;63(3):164-72. doi: 10.1016/j.jdermsci.2011.06.001. Epub 2011 Jun 22. PMID: 21726982; PMCID: PMC3152677.
45. Li X, Wang L, Nunes DP, Troxler RF, Offner GD. Suppression of MUC1 synthesis downregulates expression of the epidermal growth factor receptor. Cancer Biol Ther. 2005 Sep;4(9):968-73. doi: 10.4161/cbt.4.9.1913. Epub 2005 Sep 13. PMID: 16082192.
